# Leptin Unveiled: A Potential Biomarker for Acute Coronary Syndrome with Implications for Tailored Therapy in Patients with Type 2 Diabetes—Systematic Review and Meta-Analysis

**DOI:** 10.3390/ijms26093925

**Published:** 2025-04-22

**Authors:** Abdulrahman Ismaiel, Gaëlle Oliveira-Grilo, Daniel-Corneliu Leucuta, Nahlah Al Srouji, Mohamed Ismaiel, Stefan-Lucian Popa

**Affiliations:** 1 2nd Department of Internal Medicine, “Iuliu Hatieganu” University of Medicine and Pharmacy, 400006 Cluj-Napoca, Romania; abdulrahman.ismaiel@yahoo.com (A.I.); nahlah.alsrouji@yahoo.com (N.A.S.); popa.stefan@umfcluj.ro (S.-L.P.); 2 Faculty of Medicine, “Iuliu Hatieganu” University of Medicine and Pharmacy, 400349 Cluj-Napoca, Romania; oliveiragrilogaelle@gmail.com; 3 Department of Medical Informatics and Biostatistics, “Iuliu Hatieganu” University of Medicine and Pharmacy, 400349 Cluj-Napoca, Romania; 4 Department of General Surgery, Altnagelvin Hospital, Londonderry BT47 6LS, UK; dr.mohamed.sami.i@gmail.com

**Keywords:** acute coronary syndrome (ACS), ischemic heart disease (IHD), adipokines, leptin, non-invasive biomarkers

## Abstract

Several studies evaluated the association between adipokines, including leptin, in patients with acute coronary syndrome (ACS). Nevertheless, the results have been inconclusive and conflicting. Therefore, we assessed the pertinent published studies and evaluated the association between leptin levels and ACS. In January 2023, we conducted a comprehensive systematic search using Web of Science, PubMed, Scopus, and Embase. Using the Newcastle–Ottawa Scale, we evaluated the quality of all the articles we included. The principal summary outcome was the mean difference (MD) in leptin levels. We included 16 studies in our systematic review, 10 of which were included in meta-analysis. The MD in leptin levels was then evaluated in each subgroup: the patients with ACS versus the controls, the patients with ACS versus the patients with stable angina pectoris (SAP), and the patients with type 2 diabetes mellitus (T2DM) and ACS versus the patients without diabetes, but with ACS. Respectively, the following MDs were obtained: 10.508 (95% CI 3.670–17.346); 2.408 (95% CI −0.150–4.966); and 17.089 (95% CI 5.565–28.612). The leptin levels were significantly higher in the patients with ACS compared to the healthy controls, as well as in the patients with ACS and T2DM compared to those without T2DM. However, no statistically significant increase in leptin levels was observed when comparing the patients with ACS to those with SAP.

## 1. Introduction

Ischemic heart disease (IHD), which encompasses coronary artery disease (CAD), is the leading cause of global morbidity and mortality [1]. It manifests clinically through acute coronary syndrome (ACS) and chronic coronary syndrome [2]. IHD continues to represent a major burden on public health, with its prevalence rising in low- and middle-income countries, despite improvements in prevention and treatment in high-income nations [3].

CAD is a spectrum of diseases resulting from the gradual narrowing and hardening of the coronary arteries due to atherosclerosis. ACS, a subset of CAD, refers to conditions where blood flow to the heart is acutely reduced, leading to myocardial ischemia [4]. This includes three main conditions, ST-elevation myocardial infarction (STEMI), non-ST-elevation myocardial infarction (NSTEMI), and unstable angina [5,6]. The diagnosis of ACS is based on a combination of clinical presentation, electrocardiographic changes, and elevated cardiac biomarkers, with myocardial infarction defined as evidence of myocardial injury in the setting of acute myocardial ischemia [5].

The pathophysiology of atherosclerosis involves endothelial dysfunction, lipid accumulation, and the infiltration of inflammatory cells, including macrophages, into the vessel walls [7,8]. These processes lead to plaque formation, which may eventually rupture, triggering thrombosis and acute coronary events [9,10]. The risk factors for atherosclerosis include traditional factors, such as hypertension, dyslipidemia, smoking, and physical inactivity, but emerging factors like obesity, diabetes, and aging have become increasingly important [11,12]. Environmental stressors, including poor sleep quality, sedentary lifestyles, air pollution, and psychosocial stress, are also recognized contributors to the development and progression of ACS [13].

Obesity, a major risk factor for atherosclerosis, is characterized by excess adipose tissue, which secretes bioactive molecules known as adipokines [8,14]. These adipokines, including leptin, have been implicated in various physiological processes, such as energy balance, metabolism, and inflammation [15]. Leptin, in particular, plays a crucial role in regulating food intake and energy expenditure through its action on the hypothalamus and adipose tissues [16]. However, leptin resistance, commonly seen in obesity, leads to the impaired regulation of hunger and increased caloric intake, contributing to further weight gain and metabolic dysfunction [17,18,19].

Leptin’s involvement in the inflammatory processes underlying atherosclerosis has generated interest in its potential as a biomarker for cardiovascular disease [20]. The previous studies have shown a positive correlation between elevated leptin levels and the development of CAD [21]. However, the role of leptin in ACS specifically remains unclear, with limited and inconsistent findings. Given this gap in the literature, we conducted this systematic review and meta-analysis to evaluate the association between leptin levels and ACS, aiming to clarify its potential role as a biomarker for this condition.

## 2. Methods

This meta-analysis was written based on the Preferred Reporting Items for Systematic review and Meta-analyses (PRISMA) 2020 statement (Supplementary Materials) [22].

### 2.1. Data Sources and Search Strategy

We used four electronic databases, including Embase, PubMed, Web of Science, and Scopus, to assess observational studies evaluating leptin levels in patients with ACS. The following predetermined search string was used, ((“Leptin”[Mesh]) OR (“Leptin”[All Fields])) AND ((“Acute Coronary Syndrome”[Mesh]) OR (“Acute Coronary Syndrome”[All Fields])) for PubMed, and a similar one was used for Web of Science, Scopus, and Embase. Additionally, we carefully checked the included references of articles for any potential pertinent missing publications. On 11 October 2023, two investigators (A.I. and G.O.-G.) independently completed the literature search. Any disagreement was resolved after discussion. The duration, country, and language of the search were not restricted or filtered. First, for eligibility, the titles and abstracts were scrutinized. Then, we evaluated the complete texts of the publications that met our inclusion and exclusion requirements. One researcher (G.O.-G.) extracted the data, another (A.I.) checked them, and any inconsistencies were settled by consensus. Author names, publication year, country, sample size, ACS percentage, mean age, sex distribution, leptin measurement method and source, leptin levels, and primary study outcome were among the extracted data that were compiled. We present them in this manuscript.

### 2.2. Eligibility Criteria

The following were the criteria of inclusion for our systematic review and meta-analysis: (I) the assessment of leptin levels in patients with ACS in a cross-sectional, cohort, or case-control study; (II) leptin measured from plasma or serum; (III) ACS diagnosis according to the criteria of each study; (IV) human research without regard to gender, race, or ethnicity; (V) and studies that were written in English, French, German, or Romanian.

The following were the exclusion criteria: (I) patients with IDH that do not belong to a specific ACS group; (II) patients with stable CAD without ACS; (III) and any type of information that was not included in a full article.

### 2.3. Risk-of-Bias Assessment in Individual Studies

Using the Newcastle–Ottawa Scale (NOS), the researchers (A.I. and G.O.-G.) independently assessed the internal validity and bias risk of the included studies. Discussion was used to resolve any differences of opinion between the two investigators regarding quality assessment. Each study received a specific number of stars, which determined their score. Each included study was evaluated according to the number of stars awarded across the selection, comparability, and outcome domains. This process was vigorously established to compare the quality of each study quantitatively. Scores ranged from 0 to 10, allowing for the quantitative comparison of study quality. Studies receiving 7 or more stars were categorized as high-quality. Quality assessment did not influence the eligibility of the studies.

### 2.4. Summary Measures and Synthesis of Results

For the analyses of the data, we employed the R with Metafor package (OpenMeta [Analyst]) [23,24]. The mean difference (MD) in leptin levels was the primary summary outcome. Additionally, we evaluated research heterogeneities and inconsistencies using the Q test and I^2^ statistics. We estimated I^2^ values from 0% to 40% as not important, from 30% to 60% as moderate heterogeneity, from 50% to 90% as substantial heterogeneity, and from 75% to 100% as considerable heterogeneity according to the Cochrane Handbook for detecting and assessing heterogeneity. The random effects model and MD were employed to assess the estimated total effect size. According to the recommendations in the Cochrane Handbook, we split the groups into several subgroups. The leptin levels were compared in the “ACS patients versus controls”, the “type 2 diabetes mellitus (T2DM)-ACS patients versus non-T2DM-ACS patients”, and the “ACS patients versus SAP patients”. All study data were presented as the estimated MD with a 95% confidence interval (CI), a lower bound, an upper bound, a standard error, and a *p*-value. *p*-values < 0.05 were considered statistically significant. We only performed analysis when two or more studies with available mean, SD, or median (interquartile range [IQR]) leptin levels reported the same result.

## 3. Results

### 3.1. General Results

**Figure 1:**
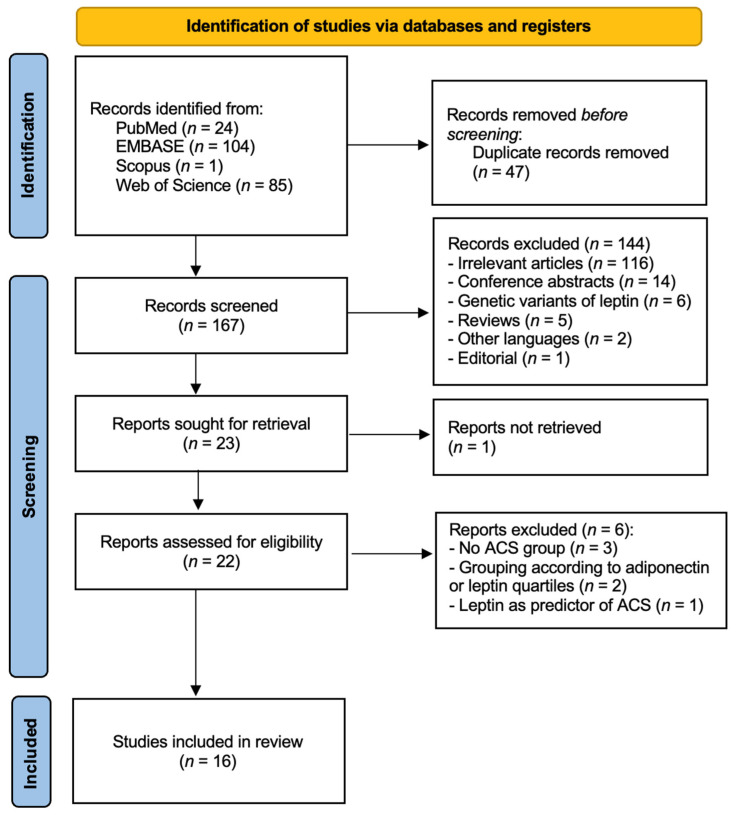
Diagram of identification, screening, and inclusion steps using PRISMA.

As shown in Figure 1, the initial search resulted in 214 articles (24 from PubMed, 104 from EMBASE, 1 from Scopus, and 85 from Web of Science). A total of 167 studies were screened after it was determined that 47 publications were duplicates and were thus eliminated. By screening the titles and the abstracts for eligibility, the remaining studies were then assessed in accordance with the inclusion and exclusion criteria. Another 151 studies were excluded, with the reasons being mentioned in Figure 1. A total of 16 studies were included in this systematic review [21,25,26,27,28,29,30,31,32,33,34,35,36,37,38,39]. After the design of three different subgroups, 10 out of the 16 studies were included in meta-analysis [21,25,27,28,32,34,36,37,38,39].

### 3.2. Study Characteristics

The main characteristics of the included studies are displayed in Supplementary Table S1. In this systematic review, 16 studies were included, with a total of 2183 subjects. A total of 10 cross-sectional studies were part of meta-analysis, involving 1735 participants, of whom 1244 (71.70%) were males and 491 (28.29%) were females. All the studies that were included reported the participants’ mean age. Three studies were conducted in Europe (Sweden [*n* = 1], Germany [*n* = 2]); six studies were conducted in Asia (China [*n* = 1], India [*n* = 2], Pakistan [*n* = 1], and Russia [*n* = 2]); and one study was conducted in the USA.

### 3.3. Leptin Evaluation

Leptin was measured in all the included studies using the enzyme-linked immunosorbent assay (ELISA) technique. Regarding the studies included in our meta-analysis, six studies used plasma samples [21,25,27,28,32,36], and the other four used serum samples for testing [34,37,38,39].

### 3.4. Leptin Levels in Patients with ACS vs. Controls

**Figure 2:**
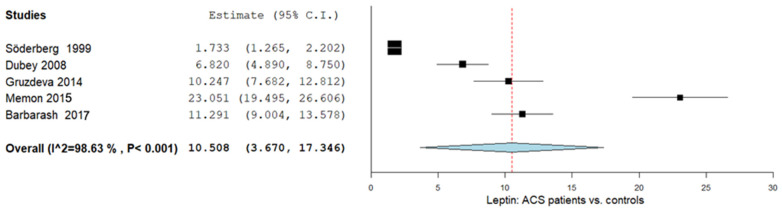
Leptin levels in patients with ACS vs. controls. ACS: acute coronary syndrome [21,28,37,38,39].

In this subgroup, five studies were included, and the serum or plasma leptin levels were measured. The leptin levels in the group whose patients were diagnosed with ACS and in the group whose subjects were healthy were then compared. The reported pooled analysis of the leptin levels resulted in an MD of 10.508 ng/mL (95% CI 3.670–17.346). We also observed significant heterogeneity with I^2^ = 98.63% and *p*-value < 0.001. The results are shown in Figure 2.

### 3.5. Leptin Levels in the Subgroup of Patients with ACS vs. Patients with SAP

**Figure 3:**
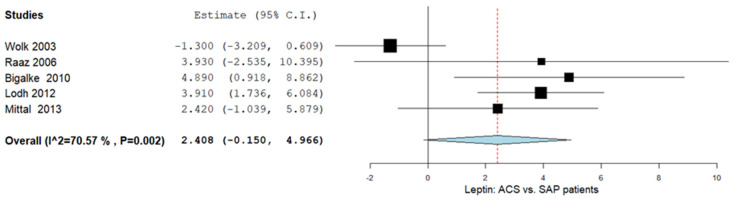
Leptin levels in patients with ACS vs. patients with SAP. ACS: acute coronary syndrome; SAP: stable angina pectoris [25,27,32,34,36].

In this subgroup, five studies were included, and the leptin levels were compared in the patients with ACS vs. the patients with stable angina pectoris (SAP). The MD of the leptin levels was 2.408 ng/mL (95% CI −0.150–4.966). Significant heterogeneity was reported with I^2^ = 70.57% and *p*-value = 0.002. The results are shown in Figure 3.

### 3.6. Leptin Levels in the Subgroup of Patients with ACS and T2DM vs. Patients Without T2DM, but with ACS 

**Figure 4:**
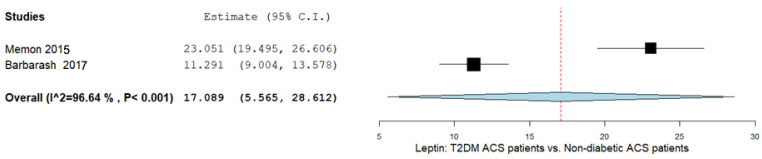
Leptin levels in patients with T2DM-ACS vs. patients without diabetes, but with ACS. T2DM: type 2 diabetes mellitus; ACS: acute coronary syndrome [38,39].

In this subgroup, leptin was assessed in two different studies, which were also included in the first group, the patients with ACS vs. the controls. The leptin levels were compared in the patients with ACS and T2DM versus in the patients with ACS without T2DM. The MD of the leptin levels in both the groups was 17.089 ng/mL (95% CI 5.565–28.612), being higher in the patients with ACS and T2DM than in the patients with ACS without T2DM. The heterogeneity reported was I^2^ = 96.64% and *p*-value < 0.001. The results are shown in Figure 4.

### 3.7. Quality Assessment

The quality of the included studies was assessed using the NOS for cross-sectional studies as outlined in Supplementary Table S2. A total of nine studies received a score of ten, while the other study received a score of eight. All the included studies that were assessed had a well-defined research topic and objectives. Every study employed carefully defined outcome measures that are regarded as valid and trustworthy. The determination of exposure was satisfactorily evaluated in nine studies.

## 4. Discussion

In high-income countries, the prevalence and fatality of CAD will probably continue to decline due to ongoing advancements in its prevention and treatment [40]. However, CAD remains the leading cause of death and reduction in Disability-Adjusted Life Years (DALYs) globally. This impacts primarily low- and middle-income nations. This places a significant economic burden on the entire world as it results in over 7 million deaths and 129 million DALYs each year [41]. Our systematic review and meta-analysis aimed to investigate the relationship between leptin levels and ACS. Our findings indicate that the leptin levels in the patients with ACS are significantly higher compared to the controls, and that the patients with coexisting ACS and T2DM exhibit even higher leptin levels than those without T2DM.

Adipokines, such as leptin, play critical roles in metabolic regulation and cardiovascular health due to their unique receptors on target cells. While adipokines like adiponectin, apelin, and omentin have anti-inflammatory and cardioprotective properties [42], others like leptin, resistin, and visfatin exert pro-inflammatory effects, contributing to cardiovascular disease [43]. Several studies have examined the relationship between adipokines and ACS. For instance, a meta-analysis demonstrated that chemerin levels were significantly higher in patients with ACS compared to the controls and notably higher in patients with ACS and T2DM [44]. These findings are consistent with our results, reinforcing the importance of inflammatory biomarkers in the pathophysiology of ACS.

Obesity is a well-established risk factor for CAD [45], and the body mass index (BMI), while commonly used, is an imperfect measure of adiposity [46]. Leptin, a key cytokine produced by adipose tissue, offers a more direct insight into obesity-related cardiovascular risk [47]. Elevated leptin levels have been linked to various metabolic disorders, including insulin resistance, T2DM, and chronic kidney disease [18]. Leptin’s role in promoting inflammation further exacerbates atherosclerosis and the risk of thrombotic events [48,49,50].

The finding that the leptin levels in the patients with ACS and T2DM are significantly higher than in those without T2DM provides valuable insights into the interplay between metabolic dysfunction and cardiovascular risk. T2DM is a major risk factor for cardiovascular diseases, including ACS, and leptin is known to influence insulin sensitivity and glucose metabolism [49]. The elevated leptin levels in the patients with ACS and T2DM may suggest that leptin could serve as both a biomarker and a potential therapeutic target for risk stratification and treatment. Further research is required to understand the underlying mechanisms of this association and to evaluate whether leptin-targeted interventions could mitigate the cardiovascular risks in these patients.

When comparing the leptin levels in the patients with ACS and the patients with SAP, we found no statistically significant difference. However, the distinction between the patients with ACS and SAP is crucial, as ACS involves acute plaque rupture and thrombosis, while SAP represents a stable form of CAD. Given the lack of significant difference in the leptin levels, it is important to consider the broader spectrum of CAD pathophysiology when evaluating leptin’s role in these conditions [51]. Additionally, the small number of studies included in our analysis, all of which were cross-sectional in nature, limits our ability to draw conclusions about causality.

Interestingly, the studies included in this review used different sample types, with six studies evaluating the leptin levels in serum, and four in plasma. The potential impact of specimen type on leptin measurement precision remains unclear, and further studies are needed to standardize the method of leptin measurement in cardiovascular research [52]. The leptin levels in the patients with ACS compared to the controls should also be evaluated further, especially considering sex and BMI differences. Leptin could be a useful biomarker to predict the occurrence of ACS, but more studies are required to establish baseline leptin levels and cut-off values. Additionally, it is important to monitor patients with ACS in the short and long term to determine how leptin levels affect prognosis.

The findings of this systematic review and meta-analysis have several clinical implications. Firstly, the elevated leptin levels in the patients with ACS suggest that leptin may play a role in the pathophysiology of ACS [53]. This finding opens avenues for future research to investigate whether interventions targeting leptin or its downstream pathways could be beneficial in ACS management. Secondly, the stratification of the patients with ACS based on the presence of T2DM and the elevated leptin levels could aid in identifying high-risk subgroups [18]. These patients may benefit from more intensive monitoring, aggressive risk factor modification, or targeted therapies aimed at mitigating the adverse cardiovascular effects associated with elevated leptin. Moreover, given leptin’s pro-inflammatory effects, it could serve as a target for therapeutic interventions aimed at mitigating the adverse cardiovascular effects of elevated leptin in ACS, and overall, patients with CAD [52]. This approach could lead to personalized treatment strategies that reduce inflammation and improve the outcomes for high-risk patients. The stratification of patients with ACS based on leptin levels, particularly those with T2DM, may guide clinical decision making and optimize the management of these patients, potentially leading to more intensive monitoring, aggressive risk factor modification, and targeted therapies.

Since it is a relatively new area of study, there are few studies on the relationship between leptin and ACS. Our qualitative and quantitative synthesis only includes a small number of studies, all of them being cross-sectional studies, thus causality cannot be inferred. Due to the lack of data in the included studies, subgroup analysis by ACS types was not feasible. Even though several studies indicated that leptin levels can vary across sexes and according to BMI, we were unable to conduct subgroup analysis comparing those variants due to the scant information in the published literature.

Despite these limitations, our review has several strengths. We conducted a comprehensive search of multiple medical databases, ensuring that our findings are based on a broad, representative sample of studies from diverse geographic and ethnic populations. The chosen studies were carried out in various locations around the world and included a variety of ethnic regions, making this analysis more representative.

## 5. Conclusions

Compared to the controls, the leptin levels were significantly higher in the patients with ACS. However, the leptin levels were not significantly different between the patients with ACS and SAP. Additionally, the patients with ACS-T2DM showed considerably higher leptin levels than the patients with ACS without T2DM. Leptin therefore has the potential to become a novel biomarker for ACS and perhaps contribute to the development of tailored therapy, particularly in patients who also have T2DM.

## Data Availability

The analyzed data were extracted from the cited original articles as outlined in Supplementary Table S1. The analyzed data can be provided on reasonable request by contacting the first or corresponding author.

## Supplementary Materials

The supporting information can be downloaded at: https://www.mdpi.com/article/10.3390/ijms26093925/s1.

## Abbreviations

ACS—acute coronary syndrome; BMI—body mass index; CAD—coronary artery disease; CI—confidence interval; CKD—chronic kidney disease; CVD—cardiovascular disease; DALYs—Disability-Adjusted Life Years; IHD—ischemic heart disease; IQR—interquartile range; LEP-R—leptin receptor; MD—mean difference; PRISMA—Preferred Reporting Items for Systematic review and Meta-analyses; SAP—stable angina pectoris; T2DM—type 2 diabetes mellitus.
