# Supplementary material for: Leptin Unveiled: A Potential Biomarker for Acute Coronary Syndrome with Implications for Tailored Therapy in Patients with Type 2 Diabetes—Systematic Review and Meta-Analysis
Source: Int J Mol Sci. 2025 Apr 22;26(9):3925. doi: 10.3390/ijms26093925 (PMC12071474; doi:10.3390/ijms26093925)
Supplement: Supplementary file 1 [file ijms-26-03925-s001.zip › Supplementary Table S1.pdf]

Supplementary Table S1. Studies evaluating leptin levels in acute coronary syndrome.

First Author / Year / Country	Study Design	Study Characteristics	Main Findings
**Söderberg S et al. / 1999 / Sweden**	Cross-sectional study	• **Population**: first-ever AMI patients and controls<br>• **Total subjects**: 186 (Cases: n = 62); (Controls: n = 124)<br>• **AMI patients**: 62 (33.3%)<br>• **Mean age (years):** (AMI = 54.7 [53.0-56.5]); (Controls = 54.5 [53.3-55.8])<br>• **Sex (males):** (AMI = 62); (Controls = 124)<br>• **Leptin measurement method**: double-antibody RIA with rabbit antihuman leptin antibodies<br>• **Leptin level (ng/ml) - median (IQR)**: AMI (*n*=62): 6.1 (5.0-7.4); Controls (*n*=124): 4.4 95%CI 4.0-4.9<br>• **Leptin measurement**: Plasma	Plasma leptin strongly predicts first-ever AMI. The data support the hypothesis that leptin has an important role in the development of cardiovascular disease in obesity.
**Wolk R et al. / 2003 / USA**	Cross-sectional study	• **Population**: patients undergoing coronary angiography: stable CAD, unstable CAD (UAP + AMI)<br>• **Total subjects**: 382 (Stable CAD *n* = 226; Unstable CAD *n* = 156)<br>• **ACS patients**: (unstable CAD): 156 (40.8%)<br>• **Mean age (years)**: (Stable CAD = 63.7 ± 9.4); (Unstable CAD = 59.7±10.4)<br>• **Sex (males):** (Stable CAD = 152 [67%]); (Unstable CAD = 114 [73%])<br>• **Leptin measurement method**: not specified<br>• **Leptin level (ng/ml) - median (IQR)**: Stable CAD (*n*=226): 9.9 (5.6-19.1); Unstable CAD (*n*=156): 8.3 (5.3-17.1)<br>• **Leptin measurement:** Plasma	In multivariate analysis, leptin was found to have an independent protective association with unstable CAD.
**Amasyali B et al. / 2006 / Turkey**	Cross-sectional study	• **Population**: patients with AMI who underwent TT<br>• **Total subject**: 41 (Group 1 plasma leptin <14ng/mL n=28; Group 2 plasma leptin > 14ng/mL n=13)<br>• **ACS patients**: 41 (100%)<br>• **Mean age (years)**: (Group 1 = 58 ± 13); (Group2 = 61 ± 10)<br>• **Sex (Male/Female)**: (Group 1 = 19/9); (Group 2 = 7/6)<br>• **Leptin measurement method**: radioimmunoassay<br>• **Leptin level (ng/ml) – median (IQR)**: Group 1 (n=28): 5.6 ± 4.2; Group 2 (n=13): 28.8 ± 11.9<br>• **Leptin measurement**: Plasma	Failure of reperfusion therapy with streptokinase was significantly higher in patient with plasma leptin concentrations ≥ 14 ng/mL (group 2) compared with patients with admission plasma leptin concentrations <14 ng/mL (group1).
**Raaz D et al. / 2006 / Germany**	Cross-sectional study	• **Population**: patients with ACS (UAP and AMI) and controls (SAP or healthy)<br>• **Total subject**: 31 (21 ACS (UAP *n* = 14; AMI *n* = 7); 10 controls (SAP or healthy n = 10)<br>• **ACS patients**: 21 (67.7%)<br>• **Mean age (years)**: (SAP = 68.4 ±9.8); (ACS = 66.8 ± 10.9)<br>• **Sex (males)**: (SAP = 70/30); (ACS = 61.9/38.1)<br>• **Leptin measurement method**: Human Leptin RIA Kit (Linco, USA)<br>• **Leptin level (ng/ml) – median (IQR)**: SAP (n=10): 7.67 ± 8 ng/ml; ACS (n=21): 11.60 ±9.7 ng/ml<br>• **Leptin measurement**: Plasma	Significant differences of Leptin values were detected between patients with ACS and patients with SAP.
**Dubey L et al. / 2008 / PR China**	Cross-sectional study	• **Population**: Patients with UA (angiographically studied) and controls (no evidence of coronary artery disease)<br>• **Total subject**: 67 (UAP n = 47 (simple lesion n = 18; complex lesion n = 29)) ; (controls n = 20)<br>• **ACS patients**:47 (70.14%)<br>• **Mean age (years)**: (Controls = 55 ± 7); (Simple lesion = 57 ± 13); (Complex lesion = 59 ± 11)<br>• **Sex (males)**: (Controls n = 15 (75%)); (Simple lesion n = 14 (77%)); (Complex lesions n = 22 (76%)<br>• **Leptin measurement method**: enzyme-linked immunosorbent assay (ELISA) kits<br>• **Leptin level (ng/ml) – median (IQR)**: Control (n=20): 5.08 ± 2.81; UAP (n=47): 11.90 ± 5.20<br>• **Leptin measurement**: Plasma	Leptin concentration is significantly higher in the UA group with complex lesions compared to UA group with simple lesion and the control group. Leptin could be an independent predictor for high risk complex lesion in UA patients.
**Dubey L et al. / 2008 / China**	Cross-sectional study	• **Population**: Patients with ACS, stable angina, and healthy controls<br>• **Total subject**: 76 (controls n = 21); (SAP n = 21); (ACS n = 34)<br>• **ACS patients**: 34 (44.73%)<br>• **Mean age (years)**: (controls = 56 ± 8); (SAP = 58 ± 10); (ACS = 57 ±11)<br>• **Sex (males)**: (controls = 15 [71%]); (SAP = 16 [76%]); (ACS = 27 [79%])<br>• **Leptin measurement method**: ELISA<br>• **Leptin level (ng/ml) – median (IQR)**: Controls (n=21): 5.14 ± 2.75; SAP (n=21): 8.97 ± 4.06; ACS (n=34): 13.36 ± 5.02<br>• **Leptin measurement**: Plasma	Plasma Leptin levels are significantly higher in the ACS and SAP group compared to the control group. Plasma There is also a significant difference between leptin levels in the SAP and ACS groups.

**Kopff B et al. / 2009 / Poland**	Cross-sectional study	• **Population**: patients with ACS who underwent PCI<br>• **Total subject**: 38<br>• **ACS patients**: 38 (100%)<br>• **Mean age (years)**: 53.34 ± 6.7 years<br>• **Sex (males)**: 38<br>• **Leptin measurement method**: immunoenzymatic method<br>• **Leptin level (ng/ml) – median (IQR)**: before rehabilitation: 835.87 ± 460.14 (724.3); after rehabilitation: 834.24 ± 450.46 (770.4)<br>• **Leptin measurement**: serum	Leptin concentrations didn't change significantly after 8 weeks of cardiac rehabilitation.
**Piestrzeniewicz K et al. / 2009 / Poland**	Cross sectional study	• **Population**: patients with STEMI treated with PCI, divided in groups according to the quartile of CRP<br>• **Total subject**: 70 (Group I <2.04 n=17); (Group II ≥ 2.04 and <3.60 n=18); (Group III ≥ 3.60 and <7.00 n=16); (Group IV ≥ 7.00 n=19)<br>• **ACS patients**: 70 (100%)<br>• **Mean age (years)**: (Group I = 53.71 ± 5.55); (Group II = 53.83 ± 6.83); (Group III = 55.06 ± 5.66); (Group IV = 52.00 ± 8.67)<br>• **Sex (males)**: 70<br>• **Leptin measurement method**: ELISA<br>• **Leptin level (ng/ml) – median (IQR)**: Group I: 21.31 ± 20.14; Group II: 29.30 ± 18.11; Group III: 34.82 ± 25.25; Group IV: 42.21 ± 20.20<br>• **Leptin measurement**: Plasma	There is a significant positive correlation between CRP and Leptin. (CRP is associated with ACS.)
**Bigalke B et al. / 2010 / Germany**	Cross-sectional study	• **Population**: patients with symptomatic CAD: ACS or SAP<br>• **Total subject**: 220 (ACS n=83); (SAP n=137)<br>• **ACS patients**: 83 (37.72%)<br>• **Mean age (years)**: (ACS = 70.4 ± 12.1); (SAP = 67.4 ± 9.5)<br>• **Sex (males)**: (ACS = 54); (SAP = 115)<br>• **Leptin measurement method**: ELISA<br>• **Leptin level (ng/ml) – median (IQR)**: ACS: 20.09 ± 16.4 ng/mL; SAP: 15.2 ± 10.9 ng/mL<br>• **Leptin measurement**: Plasma	Leptin levels were significantly higher in ACS compared with SAP.
**Barazzoni R et al. / 2012/ Italy**	Cross-sectional study	• **Population**: Patients with ACS and controls<br>• **Total subject**: 104 (ACS n=60); (Controls n=44)<br>• **ACS patients**: 60 (57.69)<br>• **Mean age (years)**: (ACS = 62 ± 11); (Controls = 62 ± 6)<br>• **Sex (males)**: (ACS=51/9); (Controls=37/7)<br>• **Leptin measurement method**: ELISA<br>• **Leptin level (ng/ml) – median (IQR)**: ACS: 6.1 ± 10.4; Controls: 6.3 ± 10.9<br>• **Leptin measurement**: Plasma	ACS patients had similar HOMA and plasma adipokines compared with controls. Obesity was associated with higher HOMA and higher leptin in both groups. In ACS patients, obesity-related changes in leptin is associated with a negative modulation of insulin resistance.
**Lodh M et al. / 2012 / India**	Cross-sectional study	• **Population**: Patients with AMI and AP<br>• **Total subject**: 167 (AMI n=105); (Controls=AP n=62)<br>• **ACS patients**: 105 (62.87)<br>• **Mean age (years)**: (AMI=56.9 ± 11.1); (Controls=48.8 ±10.9)<br>• **Sex (males)**: (AMI=86/19); (Controls=45/17)<br>• **Leptin measurement method**: ELISA<br>• **Leptin level (ng/ml) – median (IQR)**: AMI: 11.97 ± 8.5; Controls: 8.06 ± 5.8<br>• **Leptin measurement**: Serum	Serum leptin levels were significantly higher in the AMI group than in the control group.
**Günebakmaz Ö et al. / 2013 / Turkey**	Cross-sectional study	• **Population**: patients with NSTE-ACS divided in "Good collateral" and "Poor collateral"<br>• **Total subject**: 119 (Good collateral n=33); (Poor collateral n=86)<br>• **ACS patients**: 119 (100%)<br>• **Mean age (years)**: (Good collateral=61.8 ± 15.5); (Poor collateral=63.0 ± 11.5)<br>• **Sex (males)**: (Good collateral=23/10), (Poor collateral=59/27)<br>• **Leptin measurement method**: radioimmunoassay kit<br>• **Leptin level (ng/ml) – median (IQR)**: Good collateral: 4.2 (1.8-8.6); Poor collateral: 6.4 (2.4-12.6)<br>• **Leptin measurement**: serum	No association was found between serum leptin level and coronary collateral development although patients with low leptin levels are more prone to a better collateral circulation.
**Mittal A et al. / 2013 / India**	Cross-sectional study	• **Population**: patients with CAD<br>• **Total subject**: 90 (non-ACS patients n=45); (ACS patients n=45)<br>• **ACS patients**: 45 (50%)<br>• **Mean age (years)**: (non-ACS=57.49 ± 10.44); (ACS=55.58 ± 12.10)<br>• **Sex (males)**: (non-ACS=34 [75.6%]); (ACS=35 [77.81%])	Leptin levels were significantly higher in patients with ACS.

		• **Leptin measurement method**: ELISA<br>• **Leptin level (ng/ml) – median (IQR)**: Non-ACS: 9.26 ± 8.48; ACS: 11.68 ± 8.26<br>• **Leptin measurement**: plasma	
**Gruzdeva O et al. / 2014 / Russia**	Cross-sectional study	• **Population**: patients diagnosed with ST-elevation MI and controls<br>• **Total subject**: 233 (Controls n=33); (MI without IR n=46); (MI with with IR n=154)<br>• **ACS patients**: 200 (85.83%)<br>• **Mean age (years)**: (Controls=58 [51;62]); (MI without IR=58.15 [44.00;73.50]); MI with IR=59.43 [48.01;72.10]<br>• **Sex (males)**: (Controls=18[60%]); (MI without IR= 49 [63%]); (MI with IR=90 [58.41])<br>• **Leptin measurement method**: BioVendor R&D product ELISA?<br>• **Leptin level (ng/ml) – median (IQR)**: Controls: 6.98 (4.50;9.75); MI without IR 1st day: 10.82 (8.01;25.91); MI without IR 12th day: 9.20 (7.27;13.0); MI with IR 1st day: 15.44 (8.62;30.07); MI with IR 12th day: 15.62 (8.7;25.29)<br>• **Leptin measurement**: serum	At days 1 and 12 leptin levels in patients with MI and IR were 1.5-fold higher compared to the controls and 2-fold higher compared to the patients without IR.<br>In the group with MI and IR, leptin levels were increased throughout the entire hospital stay. In the group with MI but without IR, leptin levels decreased by day 12.
**Memon A. G. et al. / 2015 / Pakistan**	Cross-sectional study	• **Population**: older control subjects, older diabetic patients without complication, older diabetic patients with MI and older non-diabetic patients with MI<br>• **Total subject**: 126 (older control subjects n=31); (older diabetic patients without complication n=33); (older diabetic patients with MI n=32); (older non-diabetic patients with MI n=30)<br>• **ACS patients**: 62 (49.20%)<br>• **Mean age (years)**: not really specified. "age and weight were not significantly different in all groups".<br>• **Sex (males)**: (older control subjects=15 [48.38%]), (older diabetic patients without complication=17 [51.51%]), (older diabetic patients with MI=18 [56.25%]), (older non-diabetic patients with MI=15 [50%])<br>• **Leptin measurement method**: ELISA<br>• **Leptin level (ng/ml) – median (IQR)**: older control subjects: 12.43 ± 3.83; older diabetic patients without complication: 31.51 ± 5.23; older diabetic patients with MI: 52.18 ± 6.06; older non-diabetic patients with MI: 38.00 ± 7.20<br>• **Leptin measurement**: serum	Leptin levels were higher in diabetic and non-diabetic patient with and without MI. These findings were accentuated in the diabetic patients with MI compared with non-diabetic patients with MI.
**Barbarash O et al. / 2017 / Russia**	Cross-sectional study	• **Population**: patients with STEMI but without DM, patients with STEMI with DM, controls<br>• **Total subject**: 233 (patients with STEMI but without DM n=171); (patients with STEMI with DM n=29); (controls n=33)<br>• **ACS patients**: 200 (85.83%)<br>• **Mean age (years)**: (patients with STEMI but without DM=62.58 ± 1.2); (patients with STEMI with DM=59.5 ± 2.2); (controls not specified)<br>• **Sex (males)**: (patients with STEMI but without DM=107); (patients with STEMI with DM=23)<br>• **Leptin measurement method**: ELISA<br>• **Leptin level (ng/ml) – median (IQR)**: patients with STEMI but without DM: 14.34 (9.54; 27.67); patients with STEMI with DM: 26.29 (20.45; 29.31); controls: 6.98 (4.5; 9.75)<br>• **Leptin measurement**: serum	In patients with an imbalanced adipokine state during the acute phase of MI, the proinflammatory and thrombotic potential of blood plasma were activated. A 2.05-fold increase in leptin levels were determined on the 1st day of MI compared with the control group. Leptin showed 1.8-fold increase compared with patients without DM.
**ACS –** Acute coronary syndromes; **AMI –** Acute myocardial infarction; **BMI –** Body mass index; **CAD –** Coronary artery disease; **DM –** Diabetes mellitus; **ELISA –** Enzyme-linked immunosorbent assay; **HOMA –** Homeostasis model assessment of insulin resistance; **IR –** Insulin resistance; **MI –** Myocardial infarction; **NDM –** Non-diabetic; **SAP –** Stable angina pectoris; **TT –** Thrombolytic therapy; **UAP –** Unstable angina pectoris.			
