# Supplementary material for: Leptin Unveiled: A Potential Biomarker for Acute Coronary Syndrome with Implications for Tailored Therapy in Patients with Type 2 Diabetes—Systematic Review and Meta-Analysis
Source: Int J Mol Sci. 2025 Apr 22;26(9):3925. doi: 10.3390/ijms26093925 (PMC12071474; doi:10.3390/ijms26093925)
Supplement: Supplementary file 1 [file ijms-26-03925-s001.zip › Supplementary Table S2 - Quality assessment.pdf]

Supplementary Table S2. The Newcastle-Ottawa Scale (NOS) for assessing the quality of cross-sectional studies.

Study	Selection				Comparability	Outcome		Score
	Sample representativeness	Sample size	Non-Respondents	Ascertainment of the exposure (risk factor)	Comparability	Assessment of the outcome	Statistical test	
Soderberg et al. 1999	*	*	*	**	**	**	*	10
Wolk et al. 2003	*	*	*	**	**	**	*	10
Raaz et al. 2006	*	-	*	**	*	**	*	8
Dubey et al. 2008	*	*	*	**	**	**	*	10
Bigalke et al. 2010	*	*	*	**	**	**	*	10
Lodh et al. 2012	*	*	*	**	**	**	*	10
Barazzoni et al. 2012	*	*	*	**	**	**	*	10
Mittal et al. 2013	*	*	*	**	**	**	*	10
Gruzdeva et al. 2014	*	*	*	**	**	**	*	10
Memon et al. 2015	*	*	*	**	**	**	*	10
Barbarash et al. 2017	*	*	*	**	**	**	*	10
